# The Influence of Anti-Infective Periodontal Treatment on C-Reactive Protein: A Systematic Review and Meta-Analysis of Randomized Controlled Trials

**DOI:** 10.1371/journal.pone.0077441

**Published:** 2013-10-14

**Authors:** Ryan T. Demmer, Ludovic Trinquart, Aleksandra Zuk, Benjamin C. Fu, Josefin Blomkvist, Bryan S. Michalowicz, Philippe Ravaud, Moïse Desvarieux

**Affiliations:** 1 Department of Epidemiology, Mailman School of Public Health, Columbia University, New York, New York, United States of America; 2 Centre d'Epidémiologie Clinique, Hôpital Hôtel-Dieu, Assistance Publique-Hôpitaux de Paris, Paris, France; 3 INSERM U738, Paris, France; 4 Université Paris Descartes - Sorbonne Paris Cité, Paris, France; 5 French Cochrane Centre, Paris, France; 6 Department of Developmental and Surgical Sciences, University of Minnesota, Minneapolis, Minnesota, United States of America; 7 EHESP School of Public Health, Rennes, France; Auburn University, United States of America

## Abstract

**Background:**

Periodontal infections are hypothesized to increase the risk of adverse systemic outcomes through inflammatory mechanisms. The magnitude of effect, if any, of anti-infective periodontal treatment on systemic inflammation is unknown, as are the patient populations most likely to benefit. We conducted a systematic review and meta-analysis of randomized controlled trials (RCTs) to test the hypothesis that anti-infective periodontal treatment reduces systemic c-reactive protein (CRP).

**Methods and Findings:**

MEDLINE, EMBASE, CENTRAL and CINAHL databases were searched using sensitivity-enhancing search terms. Eligible RCTs enrolled patients with periodontal infection, compared a clearly defined anti-infective periodontal intervention (experimental group) to an “inactive control” (no periodontal intervention) or to an “active control” (lower treatment intensity than the experimental group). Mean differences in final CRP values at the earliest post-treatment time point (typically 1-3 months) between experimental and control groups were analyzed using random-effects regression. Among 2,753 possible studies 20 were selected, which included 2,561 randomized patients(median=57). Baseline CRP values were >3.0 mg/L in 40% of trials. Among studies with a control group receiving no treatment, the mean difference in CRP final values among experimental treatment vs. control groups was -0.37 mg/L [95%CI=-0.64, -0.11], (P=0.005), favoring experimental treatment. Trials for which the experimental group received antibiotics had stronger effects (P for interaction=0.03) and the mean difference in CRP final values among experimental treatment vs. control was -0.75 mg/L [95%CI=-1.17,-0.33]. No treatment effect was observed among studies using an active treatment comparator. Treatment effects were stronger for studies that included patients with co-morbidities vs. studies that included “systemically healthy” patients, although the interaction was not significant (P=0.48).

**Conclusions:**

Anti-infective periodontal treatment results in short-term modest reductions in systemic CRP.

## Introduction

Periodontal infections have been hypothesized as a risk factor for several adverse health outcomes. Observational studies have linked periodontal infection with increased risk for developing subclinical and clinical cardiovascular disease[1–5], poor glycemic control among individuals with diabetes[6–9], increased diabetes risk[10–14], adverse pregnancy outcomes[15,16], and the development of rheumatoid arthritis[17–20].

Chronic inflammation is a potential mechanism linking periodontal infection with the aforementioned systemic inflammatory outcomes. Elevated inflammatory biomarkers, such as C-reactive protein (CRP), have been consistently shown to increase the risk for clinical outcomes such as cardiovascular disease[21,22] and type 2 diabetes[23,24]. Exposure to select microbes in dysbiotic subgingival biofilms might elicit a chronic low-grade inflammatory phenotype. Therefore, anti-infective periodontal therapies that reduce exposure to subgingival pathogenic microbes are a plausible anti-inflammatory intervention. A recent American Heart Association Scientific Statement on periodontal infections and atherosclerotic vascular disease (AVD) concluded that “periodontal interventions result in a reduction in systemic inflammation”[25] based on several randomized controlled trials (RCTs) showing reductions in inflammatory markers after periodontal treatment. The Scientific Statement also recognized that “the effects of therapy on specific inflammatory markers are not consistent across studies”[25] which is supported by the current literature including several RCTs showing either no treatment effect or increased inflammatory biomarkers after periodontal treatment. The heterogeneity observed in previous studies could arise from a number of factors including study bias, variation in treatment protocols and differences in patient characteristics/co-morbidities.

Therefore, at least three critical questions remain unanswered: i) the overall magnitude of effect, if any, of anti-infective periodontal treatment on systemic inflammation and the clinical meaningfulness of these effects; ii) whether adjunctive antibiotics result in greater reductions in inflammation; iii) and whether treatment effects are greater among patients with underlying co-morbidities. These questions are important for both future research and current clinical practice.

We have conducted a systematic review and meta-analysis of randomized controlled trials to comprehensively assess the overall evidence regarding CRP effects following anti-infective periodontal treatment. We further examined whether or not the influence of treatment on CRP levels varied according to treatment protocol or patient co-morbidities. CRP is the primary outcome in the study because it is a common surrogate of systemic inflammation and the only inflammatory outcome for which clinical recommendations exist (for patients at intermediate cardiovascular disease risk)[26].

## Methods

The *a priori* review protocol was published with the International Prospective Register of Systematic Reviews (PROSPERO) under registration# CRD42011001775. This report complies with the preferred reporting items of PRISMA for systematic reviews and meta-analyses[27].

### Criteria for considering studies for this review

#### Types of studies

We included randomized controlled trials with available CRP data. There was no minimum trial duration.

#### Types of participants

Trials that enrolled adult patients with periodontal infection (i.e., gingivitis or periodontitis) were eligible.

#### Types of interventions

Eligible studies used any surgical or nonsurgical periodontal treatment with or without antibiotics or other chemical adjuncts. Scaling and root planing (SRP), a mainstay in periodontal therapy, was reported in all but one study and is defined by the American Academy of Periodontology as “a non-surgical procedure where the therapist removes plaque and calculus from the periodontal pocket and around the tooth root and smooths the root surfaces to promote healing.” Control groups were defined as

“inactive” if any of the following occurred during the same study period as the experimental group: i) there was no periodontal treatment; ii) oral hygiene instruction (OHI) was provided; iii) supragingival cleaning was performed; iv) teeth with disease so severe that treatment was not indicated were extracted. We also considered trials in which the control group received an “active” periodontal treatment with a distinctly lower treatment intensity than the experimental group, e.g., SRP + antibiotic treatment in the experimental group vs. SRP alone in the control group. Referral to “community periodontal care” was considered an active treatment protocol.

#### Types of outcome measures

The primary outcome was the CRP level after periodontal treatment because it is commonly studied as an inflammatory outcome in periodontal treatment studies and also has clinical utility[26]. The primary outcome was based on the CRP measurement at the shortest duration from treatment completion, with the requirement that at least two weeks separated treatment completion and post-treatment CRP measurement. In a secondary analysis, we considered the CRP level at 3-month follow-up, as it was the most frequently reported post-treatment time point assessed. Secondary outcomes included the local clinical measures of inflammation, bleeding on probing and probing depth, which serve as markers of anti-infective treatment success.

### Search methods for identification of studies

We searched Pubmed/MEDLINE (up to February 6^th^, 2013) and the Cochrane Oral Health Group Specialized Register, EMBASE, CENTRAL and CINAHL(up to March 2012). There was no date restriction but only publications in the English language were reviewed. The search equation for MEDLINE is reported in Table S1 in File S1. In addition we searched the references of all identified studies for more trials as well as the conference proceedings from the three most recent years of the two main meetings for the following specialties: dentistry, cardiology, obstetrics, neurology, endocrinology, rheumatology. We also searched the WHO international clinical trials register platform.

### Data collection

#### Selection of studies

Two independent reviewers blinded to the results of the other reviewer first screened all records at the title level. To enhance sensitivity, records were only removed if both reviewers excluded at the title level. The second level of review was at the abstract level followed by another round of review at the full-text level. 

#### Data extraction and management

Two independent reviewers (in duplicate) abstracted data using a standardized form. When there was disagreement it was resolved by discussion with a third review author. There were no discrepancies in the determination of CRP outcome values or bias assessments. Corresponding authors were contacted via e-mail at least three times to obtain data if the CRP outcomes could not be readily abstracted from the publication.

#### Assessment of risk of bias in selected trials

Two reviewers independently assessed risk of bias using the tool described in the Cochrane Handbook for Systematic Reviews of Interventions[28]. Risk of bias was rated as low, high or unclear regarding: i) sequence generation; ii) allocation concealment; iii) blinding; and iv) completeness of outcome data.

### Data synthesis

#### Measure of treatment effect and meta-analyses

For the primary outcome - CRP values at the earliest time point after treatment completion - we measured the treatment effect using mean difference (MD) in final values between the experimental and control groups. Negative values favor experimental group over control group. For the secondary outcomes - bleeding on probing (as a percentage) and probing depth in millimeters (mm) - the treatment effect was measured using mean differences in final values between the experimental and control groups. Trials with multiple experimental groups were included separately using a shared control group divided out approximately evenly among the comparisons. Because of the expected clinical diversity, meta-analyses were performed by using DerSimonian and Laird random-effects models. To assess heterogeneity across trials, we used forest plots as well as Cochran’s heterogeneity statistics and Higgins I^2^ coefficients. We produced funnel plots to assess small-study effects.

Active-inactive trials and active-active trials were synthesized separately. We repeated all syntheses with the measurements at 3-month follow-up. The same methods were used to synthesize data regarding bleeding on probing and probing depth.

#### Sensitivity analyses

To incorporate potential differences in baseline values within or between trials, we also analyzed change scores. For the analysis of change scores, the within-group correlation coefficient (ρ) between baseline and final values is required but was never reported. We requested, and were provided with, the original CRP data from the largest trial included in this meta-analysis[29]. Among the 620 participants from that trial, the correlation between pre- and post-treatment raw CRP values was 0.43 and the correlation between log-transformed values was 0.64. We performed a sensitivity analysis using a variety of values for ρ (0, 0.25, 0.5 and 0.75). As results were very similar and based on correlations observed in the original data provided by Michalowicz et al. [29], we report the results for ρ=0.5. We also performed a meta-regression analysis of final CRP values regressed on baseline CRP values and intervention status.

To account for the potentially skewed distribution of CRP, we performed a sensitivity analysis by transforming the data to the logarithmic scale[30]. The meta-analysis of data on the logarithmic scale gave similar results; thus we chose to present the non log transformed results to simplify interpretation.

To account for incomplete outcome data, we performed sensitivity analyses by imputing missing outcome data. First, we conservatively assumed that the mean CRP final value among patients with missing outcome data in the experimental group was 10% larger than observed for complete cases in the experimental group and that mean CRP final values among controls with missing data were 10% lower[31]. Second, we assumed that the mean CRP final value in participants with missing outcome data was equal to that in complete cases in the control group.

#### Subgroup analyses

We performed two subgroup analyses based on the use of antibiotics (periodontal interventions that included antibiotics vs. those that did not), and on the presence of comorbidities (healthy patients vs. patients with type 2 diabetes vs. patients with other comorbidities). Interaction tests were performed through meta-regression models.

All analysis were done using Stata MP version 11.0.

## Results

### Description of studies

#### Results of the search

**Figure 1:**
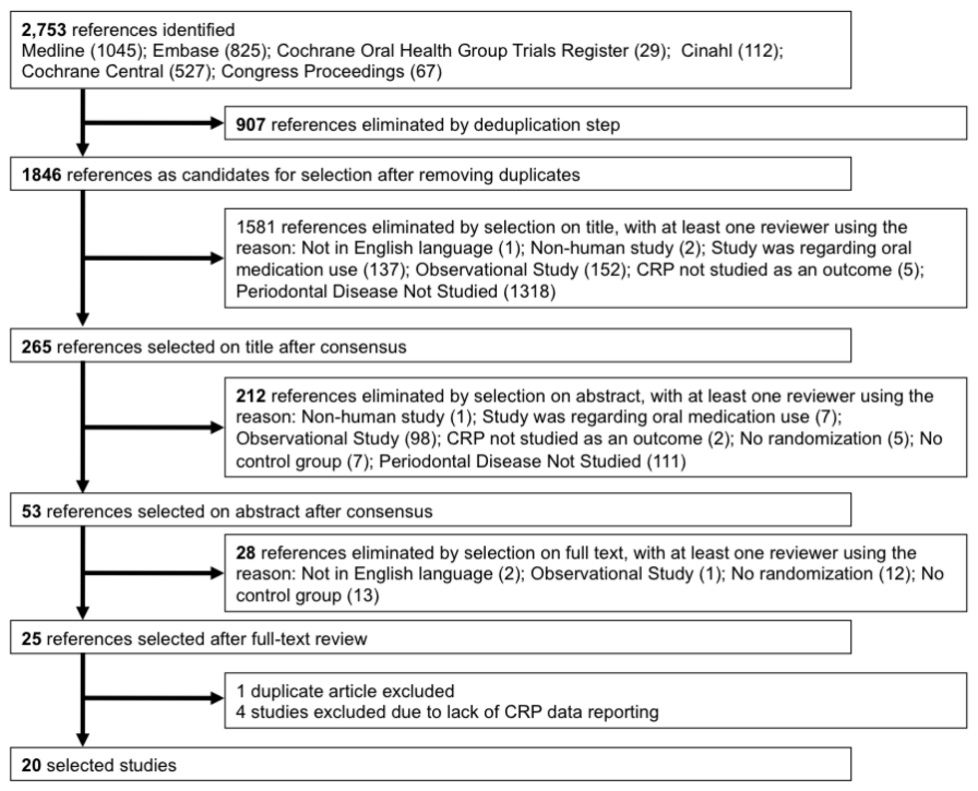
Study selection flow diagram.

Among the initial 2,753 records identified, 53 articles were reviewed at the full-text level and 25 were eligible (Figure 1). Among those, we could not exclude the possibility that two papers included a group of patients common to both studies arising from one three-arm trial[32,33]; we selected the original report[32] as the subsequent publication reported results for two arms only. Among the 24 trials, appropriate CRP data could not be abstracted from 8 trials (typically because data were presented graphically and not numerically). The corresponding and/or first authors from these 8 trials were contacted and we obtained CRP data for 4 out of 8 trials, yielding a total of 20[29,32,34–51] included trials.

#### Characteristics of included trials and interventions

Characteristics of included trials and interventions are summarized in Table 1 and in Tables S1-S2 in File S1. All but 3 trials were single-center trials. The 20 included trials were conducted across 14 different countries and 5 continents.

**Table 1:** Descriptive characteristics of 20 selected trials.

Trial size^*^	57 [12-796]
**Comparisons**	# of trials
Active periodontal treatment versus inactive control	18 randomized comparisons (16 trials)
1. SRP versus no treatment	4 [32,38,48]
2. OHI+SRP versus no treatment	6 [29,34,35,39,41,47]
3. OHI+SRP versus OHI	2 [44,49]
4. SRP+antibiotics versus no treatment	2 [32,45]
5. OHI+SRP+antibiotics versus no treatment	3 [40,43,46]
6. OHI+SRP+antibiotics versus OHI	1 [36]
Active periodontal treatment versus active treatment but with a lower intensity	6 randomized comparisons (6 trials)
1. Intensive SRP versus SRP	1 [48]
2. OHI+SRP vs. community care	1 [37]
3. SRP+antibiotic versus SRP	1 [32]
4. OHI+SRP+antibiotics versus OHI+SRP	3 [42,50,51]
**Patient characteristics**	
Age, years^†^	44 [26-60]
Female sex^†^	54% [15%-100%]
Comorbities/conditions	
Healthy	5
Pregnant	2
Diabetes	7
Other comorbidities	6
Baseline PD, mm^†^	3.4 [2.1-5.1]
Baseline % of periodontal sites with BOP^†^	54% [32.5-70.5]
Baseline CRP, mg/L^†^	3.1[0.8- 9.3]

BOP: Bleeding on probe; PD: Probing depth; SRP: Scaling and root planning;

Baseline PD, BOP and CRP values were not reported in 9, 5 and 1 trials, respectively

* Median [min-max]

† Overall mean [min-max mean values] across trials

Among the 20 selected trials, 18 were two-arm trials and 2 were three-arm trials. Among the three-arm trials, one compared SRP+antibiotics versus SRP versus no treatment, the other compared SRP at baseline and 3 months versus SRP at baseline only versus no treatment. Overall, there were 18 randomized comparisons between an active periodontal treatment and an “inactive” control group (no periodontal treatment, OHI or supragingival cleaning) and 6 randomized comparisons of an active periodontal treatment to an “active” control but with lower treatment intensity(Table 1 and Table S3 in File S1). The 4 excluded studies[52–55] (because CRP data could not be obtained) addressed comparisons of an active periodontal treatment to an active control with lower treatment intensity.

#### Characteristics of patients

The 20 selected studies included 2,561 randomized patients. The median trial size was 57 patients. All patients were adults between the ages of 26 and 60 years, with an overall mean age of 44 years; the two studies with the lowest mean patient ages enrolled pregnant women. Both genders were well represented (Table 1). Patients were frequently enrolled based on the presence of co-morbidities or pregnancy, 7 studies enrolled patients with pre-diabetes or diabetes mellitus, 6 studies enrolled patients with a variety of other co-morbidities and 2 studies enrolled pregnant women (Table 1).

All patients had periodontitis although the definitions used for inclusion varied greatly. All but two studies[45,48] required clinical evidence of current periodontal inflammation

(i.e., multiple sites with either bleeding on probing (BOP) or probing depths (PD) ≥4 mm). At baseline, the overall mean values of PD and % of periodontal sites with BOP were 3.4 mm and 54% across all studies, respectively. The median CRP value at baseline was 3.1 mg/L and 90% and 40% of study populations had a mean CRP level of ≥1.0 mg/L or >3.0 mg/L, respectively.

#### Risk of bias of included trials

**Figure 2:**
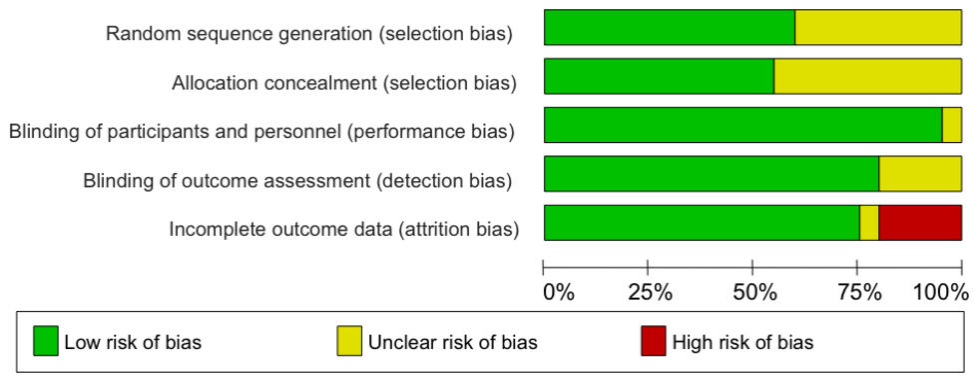
Risk of bias graph.

Overall, 10 trials had a low risk of selection bias as the sequence of generation and allocation concealment were adequate (Figure 2 and Figure S1 in File S1); in the remaining 10 trials, the risk of bias was unclear because it was not explicitly described whether sequence generation and/or allocation concealment was adequately done. Of note, 3 trials used an unequal randomization ratio of 2:1[34,36,47]. Protection against performance and detection biases was adequate as participants, personnel and outcome assessment (CRP level is objectively measured) were blinded. However, there was high risk of attrition bias in 4 trials because the proportion of patients with missing outcome data was substantial, ranging from 22% to 51%, with imbalances between the experimental and control group in 2 trials.

### Efficacy of active periodontal treatment versus inactive control on systemic CRP

**Figure 3:**
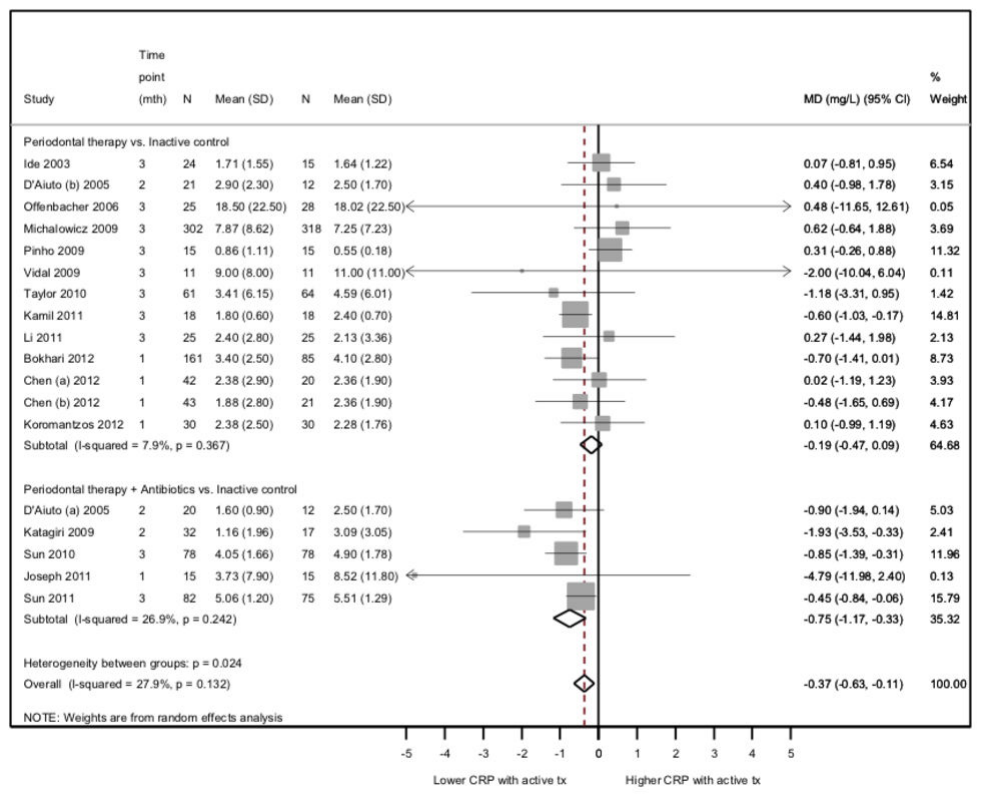
Efficacy of active periodontal therapy versus inactive control on post-treatment CRP values.

Overall, 16 trials contributed to 18 randomized comparisons between an active periodontal treatment, with or without antibiotics, and an inactive control group. The meta-analysis showed that final CRP values (earliest follow-up time point) were significantly lower, on average, in the active periodontal group than in the inactive control group (combined MD=-0.37 mg/L, 95%CI=-0.64 to -0.11, P=0.005, Figure 3). Statistical heterogeneity was moderate (Cochran’s heterogeneity test P=0.10; I^2^ coefficient=32%). 

The meta-analysis of change scores yielded stronger results (combined MD=-1.50, 95%CI=-2.11 to -0.89, P<0.001, Figure S2 in File S1), although heterogeneity was considerable. The meta-regression analysis also showed that the mean final CRP values were strongly positively associated with mean pre-treatment values, but were lower after active periodontal treatment than inactive control (mean difference in final CRP values between active periodontal treatment and inactive control, adjusted for mean baseline CRP values=-0.34 mg/L (95%CI=-0.60 to -0.10), P=0.006, Figure S3 in File S1).

A reanalysis following imputation of missing outcome data indicated that our results were robust for the 2 hypotheses considered (Figures S4-S5 in File S1). Results were also consistent when only considering studies that reported CRP outcomes at 3 months (Figure S6 in File S1).

### Subgroup Analysis

Among studies using untreated control groups, the difference in final CRP values was larger for experimental groups receiving adjunctive antibiotics (combined MD=-0.75, 95%CI=-1.17 to -0.33, with low heterogeneity) than for groups without antibiotics (combined MD=-0.19, 95%CI=-0.47 to 0.09, with low heterogeneity; P for interaction=0.03; Figure 3). Among studies with an active comparator, only 3 of 6 trials compare periodontal therapy+antibiotics to periodontal therapy alone[50,51,56] and these results show no evidence that antibiotic therapy provides stronger reductions in CRP. However, there is considerable heterogeneity between study. 

The difference in final CRP values between active periodontal treatment and inactive control was larger in patients with diabetes or another comorbidity than in healthy patients (two of which included only pregnant women), although there was no statistically significant interaction (P=0.32; Figure S7 in File S1).

Funnel plots did not show evidence of small-study bias (Figure S8 in File S1).

### Efficacy of active periodontal treatment versus active control on systemic CRP

**Figure 4:**
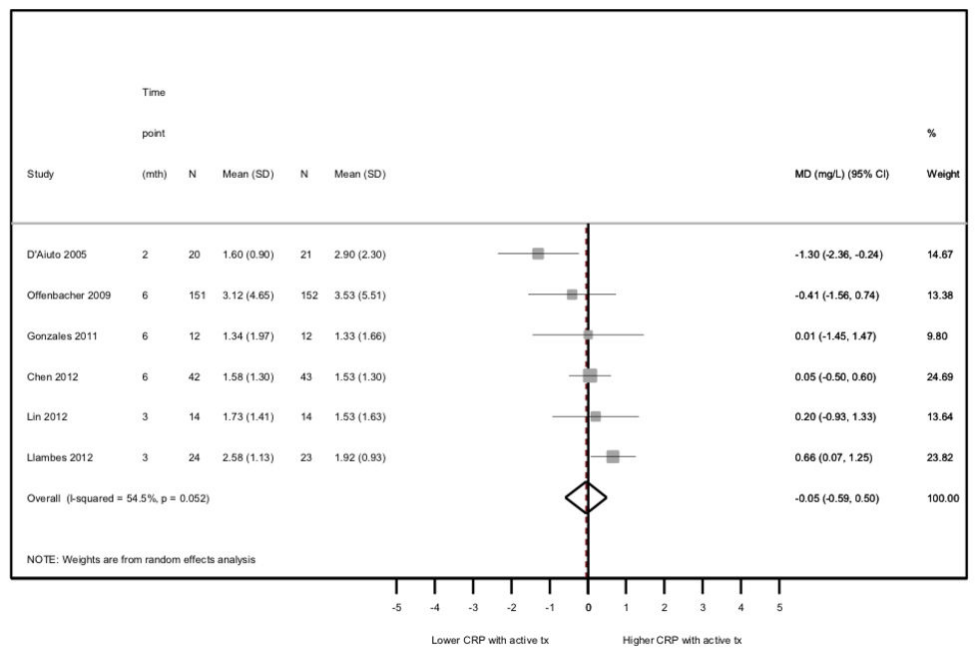
Efficacy of active periodontal therapy versus active control on post-treatment CRP values.

Overall, 6 trials contributed to comparisons between an active periodontal treatment and an active control group but with lower intensity (totaling 527 patients). The meta-analysis showed that, on average, final CRP values (earliest follow-up time point) did not differ between the two (combined MD=-0.05, 95%CI=-0.59,0.50; Figure 4), with moderate statistical heterogeneity (Cochran’s heterogeneity test P=0.05; I^2^ coefficient=55%). Among the 4 studies excluded because CRP data were not obtained[52–55], 362 patients were enrolled; one showed greater CRP reductions in the experimental group, three showed greater reductions in the control group but none reported statistically significant CRP treatment effects.

### Efficacy of experimental interventions on periodontal inflammation

Among studies reporting BOP outcomes, treatment reduced absolute BOP values by 20% on average (P<0.05; substantial heterogeneity across trials); when considering both the earliest post-intervention time point or a 3-month time point, results were notably stronger for designs using an inactive control (Figure S9 in File S1). Similarly, mean probing depth values decreased by 0.61 mm more, on average, among treated than control groups (P<0.05; substantial heterogeneity across trials) although the effect of treatment was stronger and only statistically significant for studies without an active treatment comparator (Figure S10 in File S1).

## Discussion

We have found that anti-infective periodontal treatment reduces post-treatment systemic CRP values by ~0.4 mg/L, among adult men and women. When considering periodontal interventions that included antibiotics, as compared to untreated control groups, the CRP reductions were 0.75 mg/L, although greater CRP reductions were not observed in studies using active comparators. There was also evidence to suggest that anti-infective treatment resulted in greater CRP reductions among individuals with comorbidities such as diabetes mellitus as compared to CRP reductions observed in studies enrolling apparently healthy patients although the interaction was not statistically significant.

The finding that anti-infective periodontal treatment reduces levels of an established systemic inflammatory biomarker strengthens the hypothesis that adverse exposure to microbes in dysbiotic periodontal biofilms contribute to chronic systemic inflammation. Accordingly, the current findings further strengthen the hypothesis that periodontal infections may independently contribute to the development of chronic diseases such as diabetes and cardiovascular disease since inflammation is a commonly cited biological mechanism underlying these associations[4,57].

To our knowledge, the only recommendation for use of CRP in clinical and/or public health practice was published by a joint American Heart Association (AHA)/Centers for Disease Control (CDC) working group[26]. The working group endorsed the optional use of hs-CRP in primary prevention settings to identify “patients at intermediate risk (eg, 10% to 20% risk of coronary heart disease over 10 years), in whom the physician may need additional information to guide considerations of further evaluation”. 

While it is not known whether the magnitude of CRP reductions associated with periodontal intervention in this meta-analysis can reduce clinical cardiovascular outcomes, results from two trials published subsequent to the AHA/CDC statement have demonstrated that statin therapies which reduced clinical cardiovascular outcomes also lowered CRP. In the Justification for the Use of Statins in Prevention (JUPITER) trial, patients without an LDL-cholesterol indication for statin treatment randomized to Rosuvastatin realized a reduction in median CRP values that were 1.2 mg/L greater than patients randomized to placebo. This translated into a 44% reduction in incident clinical cardiovascular events after 12 months[58]. Similarly, the Pravastatin or Atorvastatin Evaluation and Infection Therapy- Thrombolysis in Myocardial Infarction 22 (PROVE IT–TIMI 22) study[59] reported that after treatment with 40 mg of pravastatin vs. 80 mg of atorvastatin CRP levels were reduced 0.7 and 0.8 mg/L after 30 days and four months, respectively, and that these declines resulted in CVD event rate reductions equivalent to what was observed for patients who achieved LDL-cholesterol treatment goals. Therefore, CRP reductions associated with anti-infective periodontal treatment in this meta-analysis are of a similar magnitude as CRP reductions that have been associated with reduced clinical CVD event rates.

In the context of primary diabetes prevention, we are unaware of trials that have investigated CRP reduction for the prevention of diabetes development. However, high-quality observational data from the Women’s Health Study offer some insights. Pradhan et al. reported that during a four-year follow up period, elevated baseline CRP was associated with a 30% increase in the risk for incident diabetes when comparing participants in the second vs. first quartile of CRP, which corresponded to a difference in median CRP values of 1.2 mg/L[23]. Elevated CRP has been recently shown in a systematic review and meta-analysis to be associated with an ~30% increase in the risk of diabetes development[60]. The results were based on ten prospective studies including >19,000 participants and >4,000 diabetes cases.

From a secondary diabetes prevention standpoint, there is evidence from randomized controlled trials that anti-infective periodontal treatment improves glycemic control among patients with diabetes[61] and the current findings offer insights as to the possible biological mechanisms underlying those findings. Of note, in accordance with our current results, periodontal treatment trials targeting glycemic control among diabetics have also demonstrated stronger treatment effects when antibiotics are used[61].

If anti-infective periodontal treatment can be demonstrated to not only reduce systemic inflammation but also prevent adverse chronic disease outcomes it would be of high public health and clinical importance for at least two reasons. First, periodontal interventions have been shown to be safe[62,63] and often do not require pharmacological agents thus reducing the potential for adverse events and offering new treatment options for individuals with contraindications for anti-inflammatory medications such as statins or aspirin. The fact that recent evidence suggests a very modest increase in diabetes risk among statin users[64] is also a reason to possibly consider alternate anti-inflammatory treatment modalities among patients at intermediate CVD risk[26]. Moreover, because periodontal treatment is necessary for the maintenance of good oral health among patients with periodontitis, referring patients for periodontal evaluation, and possible treatment, is a reasonable first step in managing inflammatory burden among at risk patients.

Beyond their clinical and public health implications, the current findings also offer some useful insights for future research endeavors that are necessary to test whether or not anti-infective periodontal treatment can prevent the development of clinical outcomes such as diabetes and cardiovascular disease. Studies using “active control” groups that receive a lower intensity treatment regimen than the experiment treatment failed to demonstrate CRP reductions. This was possibly the result of small differences in realized levels of exposure to adverse microbial species (the treatment target) between groups that each received periodontal therapy. This notion is supported by the fact that designs using “low-intensity” treatments protocols in the control arm also reported much weaker reductions in clinical periodontal outcomes (i.e., probing depth and bleeding on probing). Consequently, future trials may need to be optimized via innovative designs that allow for an untreated control group so long as appropriate data safety and monitoring plans are in place to ensure patient safety.

Two previous meta-analyses have been published regarding the contribution of periodontal infection to systemic inflammation[65,66]. While these reports were informative they were limited in a few important ways. Ioannidou et al. reported a mean difference of CRP change between experimental and control groups from only two studies[32,67] one of which was not an RCT and post-treatment CRP was assessed on the same day[67]. In a second meta-analysis, Paraskevas et al. concluded that periodontal treatment significantly reduced CRP levels although only two studies[56,68] were included in the analysis that yielded a statistically significant 0.5 mg/L CRP reduction and neither of these two studies were randomized controlled trials.

The majority of studies identified in this review enrolled patients with mean CRP levels below the high risk AHA/CDC category (3.0 mg/L) which might have impacted the strength of findings since a certain threshold of inflammation might be necessary before anti-infective/inflammatory treatments can be useful[21]. However, the results demonstrate that CRP reductions were realized regardless of baseline CRP levels suggesting that anti-infective treatments can reduce inflammation even among participants with CRP<3.0 mg/L.

As with any meta-analysis, we cannot rule out the possibility that exclusion of unpublished trials or lack of data access and loss-to-follow-up among published trials might have biased our results towards a positive finding. The fact that there was no evidence of publication bias in addition to the consistent results showing treatment benefit arising from conservative sensitivity analyses substantially mitigates these concerns. Our attempts to obtain original data from studies for which results could not be abstracted also minimize the potential for bias. Another possible bias in this research area is the difficulty in using placebo controlled double-blind studies, which could result in overestimates of treatment effects. For example, patients who know they are receiving treatment might be more motivated to make other lifestyle changes (smoking cessation, dietary changes etc.) that could influence inflammatory outcomes.

We observed evidence of heterogeneity in treatment effects across studies which might be accounted for by a number of factors such as severity of baseline infection or CRP levels, different underlying patient co-morbidities or medications, or variation in treatment modalities. In regard to the latter, it is notable that heterogeneity was minimized when analyzing subgroups with more homogenous treatment protocols (e.g., periodontal treatment + antibiotics vs. control groups without any treatment).

Anti-infective periodontal therapy among adult men and women with periodontitis results in modest short-term systemic CRP reductions based on results from 20 randomized controlled trials conducted in 14 different countries across 5 continents. Whether or not these CRP reductions can translate into decreased rates of clinical cardiovascular disease or diabetes development remains to be determined in future clinical trials as no such trial has been conducted to date. Until such trials are performed, the evidence suggests that among patients with periodontal infection, periodontal treatment can confer the dual advantage of improved oral health and short-term reduction in systemic inflammatory activity.

## Supporting Information

Checklist S1 (DOC)

File S1 (DOCX)
