# Supplementary material for: The Influence of Anti-Infective Periodontal Treatment on C-Reactive Protein: A Systematic Review and Meta-Analysis of Randomized Controlled Trials
Source: PLoS One. 2013 Oct 14;8(10):e77441. doi: 10.1371/journal.pone.0077441 (PMC3796504; doi:10.1371/journal.pone.0077441)
Supplement: File S1 — (DOCX) [file pone.0077441.s002.docx]

**SUPPLEMENTAL TABLES**

**Table S1. MEDLINE/PUBMED search equation**

| 1. Randomized Controlled Trials |
| --- |
| (randomized controlled trial [pt]OR controlled clinical trial [pt] OR randomized [tiab] OR placebo [tiab] OR drug therapy [sh] OR randomly [tiab] OR trial [tiab] OR groups [tiab]) NOT (animals [mh] NOT humans [mh]) |
| 2. Periodontal Interventions |
| ("Periodontal Diseases"[Mesh]) NOT ("Gingival Neoplasms"[Mesh] OR "Tooth Migration"[Mesh]) OR "Periodontium"[Mesh] OR "Periodontics"[Mesh] OR "Dental Plaque"[Mesh] OR "Oral Hygiene"[Mesh] OR "Oral Health"[Mesh] OR "Dental Care for Chronically Ill"[Mesh] OR "Alveolar Bone Losses"[Title/Abstract] OR "Alveolar Bone Loss"[Title/Abstract] OR "Alveolar Process Atrophy"[Title/Abstract] OR "Alveolar Process Atrophies"[Title/Abstract] OR "Alveolar Resorption"[Title/Abstract] OR "Alveolar Resorptions"[Title/Abstract] OR "Alveolar Bone Atrophy"[Title/Abstract] OR "Alveolar Bone Atrophies"[Title/Abstract] OR "Alveolar Process"[Title/Abstract] OR "Dental Cementum"[Title/Abstract] OR "Dental Plaque"[Title/Abstract] OR "Dental Prophylaxis"[Title/Abstract] OR "Dental Scaling"[Title/Abstract] "Epithelial Attachment"[Title/Abstract] OR Epuli*[Title/Abstract] OR "Furcation Defects"[Title/Abstract] OR "Furcation Defect"[Title/Abstract] OR Gingiv*[Title/Abstract] OR Granuloma*[Title/Abstract] AND Giant[Title/Abstract] AND Cell*[Title/Abstract] OR Gum*[Title/Abstract] OR Oral[Title/Abstract] OR Paradent*[Title/Abstract] OR Parodont*[Title/Abstract] OR Periapical[Title/Abstract] OR Pericementiti*[Title/Abstract] OR Pericoroniti*[Title/Abstract] OR Peri-Implantitis[Title/Abstract] OR Peri-Implantitides[Title/Abstract] OR Periimplantiti*[Title/Abstract] OR Periodont*[Title/Abstract] OR Pyorrhea-Alveolaris[Title/Abstract] OR "Radicular Cyst"[Title/Abstract] OR "Radicular Cysts"[Title/Abstract] OR "Root Planing"[Title/Abstract] OR "Tooth Socket"[Title/Abstract] OR Subgingiv*[Title/Abstract] OR "Tooth Loss"[Title/Abstract] OR "Teeth Loss"[Title/Abstract] OR "Tooth Losses"[Title/Abstract] OR "Teeth Losses"[Title/Abstract] OR "Tooth Mobilities"[Title/Abstract] OR "Teeth Mobilities"[Title/Abstract] OR "Teeth Mobility"[Title/Abstract] OR "Tooth Mobility"[Title/Abstract] |
| 3. C-reactive protein |
| "C-Reactive Protein"[Mesh] OR “C-Reactive Protein”[Title/Abstract] OR “Protein C-Reactive”[Title/Abstract] OR “CRP”[Title/Abstract] |
| Equation: 1 AND 2 AND 3 |

**Table S2. Descriptive characteristics of selected trials**

| **First Author** | **Year** | **No centers** | **Country** | **Sample size** | **Mean age [Range]** | **% Female** | **Comorbidities/**  **Conditions** | **Periodontal inclusion criteria** | **Baseline PD (mm)** | **Baseline BOP (%)** |
| --- | --- | --- | --- | --- | --- | --- | --- | --- | --- | --- |
| Offenbacher | 2006 | 1 | US | 74 | 26 [>17] | 100% | Pregnancy | 1) ≥20 teeth; 2) ≥2 periodontal sites with ≥5 mm PD; 3) AL of 1-2 mm at 1 or more sites with ≥5 mm PD | 2.1 | 45.9 |
| Pinho | 2009 | 1 | Brazil | 75 | 50  [35-60] | 60% | Rheumatoid Arthritis | 1) ≥2 teeth with ≥6 mm AL; 2) ≥1 tooth with ≥5 mm PD | 3.2 | 41.5 |
| Vidal | 2009 | 1 | Brazil | 22 | 49  [39-62] | 50% | Severe primary refractory arterial hypertension | 1) ≥12 teeth; 2) ≥4 periodontal sites with ≥4 mm PD; 3) ≥5 periodontal sites with ≥6 mm AL | NR | 44.5 |
| Sun | 2010 | 1 | China | 156 | 57  [36-68] | 42% | 32% impaired glucose tolerance, 68% type 2 diabetes | 1) ≥20 teeth with ≥5 mm PD and ≥30% of teeth with ≥4 mm AL; or 2) ≥60% of teeth with ≥4 mm PD and ≥3 mm AL | NR | NR |
| Taylor | 2010 | 1 | Australia | 125 | 54 [NR] | 51% | 33% hyperlipidemia, 14% diabetes, 22% hypertension | 1) ≥6 periodontal sites (non-third molar sites) with ≥5 mm PD and ≥2 mm AL | 3.5 | 38.7 |
| Joseph | 2011 | 1 | India | 30 | 37 [NR] | 100% | Idiopathic edema | 1) ≥10 teeth; 2) Satisfying ≥2 of the following criteria: i) ≥1 tooth with ≥4 mm PD; ii) ≥4 teeth with ≥3 mm AL; iii) Turesky-Gillmore-Glickman modification of Quigley-Hein plaque index values >1; iv) simplified calculus index >1; v) modified gingival index values ≥1 | NR | NR |
| Kamil | 2011 | 1 | Jordan | 36 | 46  [41-53] | 44% | None reported | 1) ≥20 teeth; 2) ≥6 teeth with ≥6 mm PD and ≥3 mm AL in 3 sites of each involved tooth | NR | NR |
| Li | 2011 | 1 | Hong Kong | 50 | 59 [NR] | 45% | None reported | Any of the following: 1) more than six sites with ≥4 mm PD; or 2) >25% of sites with ≥5 mm interproximal AL; or 3) ≥9 missing teeth due to periodontitis (excluding third molars) | NR | 45.3 |
| Sun | 2011 | 1 | China | 157 | 55  [36-70] | 57% | Uncontrolled type 2 diabetes | 1) ≥20 teeth with ≥6 mm PD and ≥30% of teeth with ≥5 mm AL; or 2) ≥60% of teeth with ≥5 mm PD and ≥4 mm AL | 4.5 | NR |
| Bokhari | 2012 | 1 | Pakistan | 317 | 49  [>30] | 15% | Coronary Heart Disease | 1)≥14 teeth (excluding third molars); 2) ≥4 teeth with ≥1 site with ≥4 mm PD and >=3 mm AL at same site; 3) > 20% of sites with BOP; 4) no immediate dental/ periodontal treatment needs (e.g. extractions); and no recent (<2 months) tooth extractions | 3.5 | 41.0 |
| Ide | 2003 | 1 | UK | 39 | 47  [30-60] | 41% | None reported | 1) ≥20 teeth; 2) no untreated periapicalleasions; 3) ≥5 teeth with ≥5 mm PD and radiographic evidence of alveolar bone loss | NR | 43.1 |
| Katagiri | 2009 | 5 | Japan | 49 | 60  [39-75] | 45% | Type 2 diabetes | 1) ≥11 teeth; 2) ≥2 sites with ≥4 mm PD | 2.9 | 32.5 |
| Michalowicz | 2009 | 4 | US | 796 | 26  [16-44] | 100% | Pregnancy | 1) ≥20 teeth; 2) ≥4 teeth with ≥4 mm PD and ≥2 mm AL; 3) 35% of sites with BOP | NR | 69.3 |
| Koromantzos | 2012 | 1 | Greece | 60 | 60  [40-75] | 45% | Type 2 diabetes | 1) ≥16 teeth; 2) ≥8 periodontal sites with ≥6 mm PD; 3) 4 sites with ≥5 mm AL in ≥2 quadrants | NR | 70.5 |
| Offenbacher | 2009 | 5 | US | 303 | 60 [NR] | 29% | Coronary Heart Disease | 1) ≥6 teeth; 2) ≥3 teeth with ≥4 mm PD; 3) ≥2 teeth with ≥2 mm AL; 4) ≥10% of periodontal sites with BOP | 2.7 | 47.7 |
| Gonzales | 2011 | 1 | Germany | 12 | NR  [35-65] | 50% | None reported | 1) ≥12 teeth with ≥5 mm PD and BOP | NR | NR |
| Lin | 2012 | 1 | Taiwan | 28 | 58 [NR] | 71% | Uncontrolled type 2 diabetes | 1) ≥20 teeth; 2) ≥5 teeth with ≥5 mm PD | 5.1 | 40.5 |
| Llambés | 2012 | 1 | Spain | 53 | 35  [19-69] | 47% | Type 1 diabetes | 1) ≥14 teeth; 2) ≥5 teeth with PD≥5 mm and AL≥ 3 mm. | 3.4 | 65.0 |
| Tonetti | 2007 | 1 | UK | 120 | 47 [NR] | 50% | None reported | 1) ≥50% of dentition with ≥7 mm PD and marginal alveolar bone loss of >30% | NR | 67.0 |
| Tüter | 2007 | 1 | Turkey | 36 | 54 [<70] | 8% | Stable Coronary Heart Disease | 1) radiographic evidence of bone loss and AL; 2) ≥6 sites with ≥5 mm PD | NR | NR |
| Tüter | 2010 | 1 | Turkey | 41 | 44 [NR] | 46% | None reported | 1) radiographic evidence of bone loss and AL; 2) ≥6 sites with ≥5 mm PD | 4.2 | NR |
| López | 2012 | 1 | Chile | 165 | 55  [35-65] | 72% | Metabolic syndrome | 1) ≥14 teeth; 2) ≥4 teeth with ≥4 mm PD and ≥3 mm AL | NR | 51.5 |
| D’Aiuto | 2005 | 1 | UK | 45 | 48  [44-52] | 42% | None reported | 1) ≥50% of dentition with ≥6 mm PD and >30% alveolar bone loss | 4.5 | 67.4 |
| Chen | 2012 | 1 | China | 134 | 60 [NR] | 48% | Type 2 diabetes | 1) ≥16 teeth; 2) ≥1 mm AL on any tooth^*^; 3) mean AL ≥1 mm | 2.6 | 34.7 |

PD: Probing Depth; AL: Attachment Loss; BOP: bleeding on probe; NR: not reported

^*^Authors stated that “participants had to have a clinical diagnosis of chronic periodontitis according to the American Academy of Periodontology (AAP) criteria. Based on AAP criteria, we summarized the lowest threshold of disease that could be defined as chronic periodontitis (localized or generalized).

**Table S3. Intervention characteristics and primary outcome availability in selected trials**

| **First author** | **Year** | **Experimental intervention** | **Control intervention** | **Treatment duration** | **CRP data abstraction*** | **Mean Baseline CRP (mg/L)** | **CRP**  **method** | **Follow-up CRP**  **timepoints^†^** |
| --- | --- | --- | --- | --- | --- | --- | --- | --- |
| ***Active periodontal treatment versus Inactive control*** | | | | | | | | |
| Offenbacher | 2006 | Sonic toothbrush + OHI + SRP | Manual toothbrush without OHI + supragingival cleaning | NR | A | 8.2 | N | ~3 months^‡^ |
| Pinho | 2009 | SRP | None | NR | A | 0.8 | N** | 3, 6 months |
| Vidal | 2009 | OHI + SRP | None | 4-6 sessions during 2 weeks | A | 1.0 | N | 3 months |
| Sun | 2010 | Extractions^§^ + OHI + SRP + FS when indicated + oral antibiotics (Tinidaozole& Ampicillin) | None | NR | A | NR | IT | 3 months |
| Taylor | 2010 | Extractions^§^ + OHI + SRP | Extractions^§^ | <3 months | A | 4.6 | E | 3 months^‖^ |
| Joseph | 2011 | Extractions^§^ + OHI + SRP + chlorhexidine mouthwash + oral doxycycline | None | < 2 weeks | A | 9.3 | T | 1 month |
| Kamil | 2011 | OHI + SRP + professional plaque control every 2 months | OHI | 2-3 sessions over 10 days | A | 2.3 | IT | 3 months |
| Li | 2011 | Extractions^§^ + SRP + chlorhexidine gel in PP≥4 mm + chlorhexidine-based mouth wash for home use | None | 2-3 sessions within 1 week | A | 2.5 | IT | 3 months |
| Sun | 2011 | Extractions^§^ + OHI + SRP + FS when indicated + oral antibiotics (Tinidaozole& Ampicillin) | None | NR | A | 5.8 | IT | 3 months |
| Bokhari | 2012 | OHI + SRP | None | 2-4 visits completed within 10 days | A | 4.3 | IN | 1, 2 months |
| Ide | 2003 | OHI + SRP | None | 5 sessions in 5 weeks | B | 1.6 | IN | 3 months |
| Katagiri | 2009 | OHI + SRP + local antibiotics (minocycline) | OHI | 4 sessions over 2 months; additional OHI and SRP 1, 3 and 6 months post-intervention | B | 2.0 | IN | 1, 3, 6 months |
| Michalowicz | 2009 | OHI + SRP | None | Up to 4 visits in 5 weeks | B | 9.3 | E | 3 months |
| Koromantzos | 2012 | Extractions^§^+ OHI + SRP | OHI + supragingival cleaning | 2 visits separated by 1 week | B | 2.5 | E | 1, 3, 6 months |
| ***Active periodontal treatment versus active treatment but with a lower intensity*** | | | | | | | | |
| Offenbacher | 2009 | Extractions^§^ + OHI + SRP | Extractions^§^ + OHI + referral to seek community care | Up to 4 sessions within 2 months | A | 3.2 | N | 6, 12 months |
| Gonzales | 2011 | OHI + SRP + chlorhexidine chips | OHI + SRP+ placebo chlorhexidine chips | 3 sessions over 24 days | A | 2.5 | N | 6 months |
| Lin | 2012 | OHI + SRP followed by 1 month without tx and then 1 month of subgingival antibiotics (minocycline) | OHI + SRP | 12 weeks total: 4 SRP sessions over 1 month followed by 4 weeks without tx, concluding with 4 weeks of local antibiotic tx | A | 1.5 | E | 3, 6 months |
| Llambés | 2012 | OHI + SRP + Chlorhexidine rinses + doxycycline 100 mg B.I.D. for the first day and then one capsule per day thereafter for 15 days | OHI + SRP + Chlorhexidine rinses | 1 or 2 sessions separated by 1 week. | A | 2.3 | T | 3 months |
| Tonetti | 2007 | Extractions^§^+ OHI + SRP + local antibiotics (minocycline) | OHI + supragingival clearning + community care | 1 session | C | 3.1 | IT | 1, 2, 6 months |
| Tüter | 2007 | OHI + SRP + SDD (Periostat) | OHI + SRP | SRP during 2 sessions over 2 weeks; SDD BID x 6 weeks | C | NR | N | 2 months |
| Tüter | 2010 | OHI + SRP + SDD (Periostat) | OHI + SRP | SRP during 2 sessions over 2 weeks; SDD BID x 6 weeks | C | NR | N | 2 months |
| López | 2012 | Extractions^§^ + OHI with toothbrushes + oral Metronidazole + Amoxicillin TID x 7 days followed by SRP | Extractions^§^+ OHI with toothbrushes + SRP + placebo tables | Up to 4 weeks | C | 4.4 | E | 3, 6 months |
| ***Three-arm trials*** | | | | | | | | |
| D’Aiuto | 2005 | SRP + local antibiotics (minocycline) | None | 1 session | A | 2.4 | IT | 2 months |
|  |  | SRP |  |  |  |  |  |  |
| Chen | 2012 | SRP at baseline | None | 1 session | A | 3.0 | IT | 1, 3, 6 months |
|  |  | SRP at baseline and 3 months |  | 2 sessions, at baseline and 3 months |  |  |  |  |
|  |  |  |  |  |  |  |  |  |

SRP: scale and root planning; OHI: oral hygiene instruction; SDD: Subantimicrobial dose doxycycline; FS: flap surgery; NR: not reported

*A: original data available for abstraction in the published paper; B: original data not available for extraction in the published paper, request sent to author and data provided; C: original data not available for extraction in the published paper, request sent to author and data not provided (excluded from the meta-analysis).

^†^Unless otherwise noted, studies with interventions requiring more than one treatment visit calculated the duration of follow-up for CRP assessment(s) based on the difference between date of treatment completion and date of follow-up blood collection(s) for the intervention group.Studies that include a treated control group defined follow-up time as the difference between treatment completion and blood collection(s).Follow-up duration among untreated control groups are computed as the difference between date of randomization and date of follow-up blood collection(s).

^‡^Precise follow-up time was not reported. Participants were enrolled <22 weeks gestation and follow-up blood collections occurred at delivery (34% of participants delivered before 37 weeks of gestation). We classified this follow-up time period for this study to be 3 months.

^§^Tooth extractions as necessary

^‖^Intervention required more than one visit and the duration of follow-up for CRP assessment(s) was based on the difference between date of initial treatment and date of follow-up blood collection(s).

Method: E=ELISA, IN=immunonephelometric, IT=immunoturbidimetric, N=nephelometric, T=turbidimetric,

**Whether or not CRP assay was highly sensitive could not be determined

**SUPPLEMENTAL FIGURES**

Figure S1: Risk of bias summary (judgments about each risk of bias item for each included trial)

Figure S2: meta-analysis of CRP change scores (from baseline to earliest post-treatment measurement)

Figure S3: meta-regression analysis of mean follow-up CRP values (earliest post-treatment measurement) against mean baseline CRP values by treatment group. Red indicates treatment group and blue indicates control groups.

Figure S4: sensitivity analysis to missing outcome data - mean final CRP values (earliest post-treatment measurement) in patients of the experimental group (resp. control group) with missing outcome data assumed 10% larger (resp.,10% smaller) as compared to complete cases in the experimental group (resp., in the control group)

Figure S5: sensitivity analysis to missing outcome data - mean final CRP values (earliest post-treatment measurement) in patients with missing outcome data assumed equal to that in complete cases in the control group

Figure S6: meta-analysis of final CRP values (3-month post-treatment measurement)

Figure S7: subgroup analysis by co-mordidity (mean final CRP values at earliest post-treatment measurement)

Figure S8: funnel plot for the meta-analysis of final CRP values (earliest post-treatment measurement)

Figure S9: meta-analysis of final BOP values (earliest post-treatment measurement)

Figure S10: meta-analysis of final PD values (earliest post-treatment measurement)

**Figure S1: Risk of bias summary (judgments about each risk of bias item for each included trial)**

**Figure S2: Meta-analysis of CRP change scores (from baseline to earliest post treatment measurement)**

**Figure S3: Meta-regression analysis of mean follow-up CRP values (earliest post treatment measurement) against mean baseline CRP values by treatment group**

**Figure S4: Sensitivity analysis to missing outcome data - mean final CRP values (earliest post treatment measurement) in patients of the experimental group (resp. control group) with missing outcome data assumed 10% larger (resp.,10% smaller) as compared to complete cases in the experimental group (resp., in the control group)**

**Figure S5: Sensitivity analysis to missing outcome data - mean final CRP values (earliest post treatment measurement) in patients with missing outcome data assumed equal to that in complete cases in the control group**

**Figure S6: Meta-analysis of final CRP values (3-month follow-up)**

**Figure S7: Subgroup analysis by co-morbidity (mean final CRP values at earliest post treatment measurement)**

**Figure S8: Funnel plot for the meta-analysis of final CRP values (earliest post treatment measurement)**

**Figure S9: Meta-analysis of final BOP values (earliest post treatment measurement)**

**Figure S10: Meta-analysis of final PD values (earliest post treatment measurement)**
